# Modelling age-heterogeneous *Schistosoma haematobium *and *S. mansoni *survey data via alignment factors

**DOI:** 10.1186/1756-3305-4-142

**Published:** 2011-07-20

**Authors:** Nadine Schur, Jürg Utzinger, Penelope Vounatsou

**Affiliations:** 1 Department of Epidemiology and Public Health, Swiss Tropical and Public Health Institute, P.O. Box, CH-4002 Basel, Switzerland; 2 University of Basel, P.O. Box, CH-4003 Basel, Switzerland

## Abstract

**Background:**

Reliable maps of the geographical distribution, number of infected individuals and burden estimates of schistosomiasis are essential tools to plan, monitor and evaluate control programmes. Large-scale disease mapping and prediction efforts rely on compiled historical survey data obtained from the peer-reviewed literature and unpublished reports. Schistosomiasis surveys usually focus on school-aged children, whereas some surveys include entire communities. However, data are often reported for non-standard age groups or entire study populations. Existing geostatistical models ignore either the age-dependence of the disease risk or omit surveys considered too heterogeneous.

**Methods:**

We developed Bayesian geostatistical models and analysed existing schistosomiasis prevalence data by estimating alignment factors to relate surveys on individuals aged ≤ 20 years with surveys on individuals aged > 20 years and entire communities. Schistosomiasis prevalence data for 11 countries in the eastern African region were extracted from an open-access global database pertaining to neglected tropical diseases. We assumed that alignment factors were constant for the whole region or a specific country.

**Results:**

Regional alignment factors indicated that the risk of a *Schistosoma haematobium *infection in individuals aged > 20 years and in entire communities is smaller than in individuals ≤ 20 years, 0.83 and 0.91, respectively. Country-specific alignment factors varied from 0.79 (Ethiopia) to 1.06 (Zambia) for community-based surveys. For *S. mansoni*, the regional alignment factor for entire communities was 0.96 with country-specific factors ranging from 0.84 (Burundi) to 1.13 (Uganda).

**Conclusions:**

The proposed approach could be used to align inherent age-heterogeneity between school-based and community-based schistosomiasis surveys to render compiled data for risk mapping and prediction more accurate.

## Background

An estimated 200 million individuals are infected with *Schistosoma *spp. in Africa, and yet schistosomiasis is often neglected [1]. The global strategy to control schistosomiasis and several other neglected tropical diseases (NTDs) is the repeated large-scale administration of anthelminthic drugs to at-risk populations, an approach phrased 'preventive chemotherapy' [2,3]. The design, implementation, monitoring and evaluation of schistosomiasis control activities require knowledge of the geographical distribution, number of infected people and disease burden at high spatial resolution.

In the absence of contemporary surveys, large-scale empirical risk mapping heavily relies on analyses of historical survey data. For example, Brooker *et al*. [4] compiled survey data and presented schistosomiasis (and soil-transmitted helminthiasis) risk maps within the global atlas of helminth infections (GAHI) project (http://www.thiswormyworld.org/). The GAHI database, however, is not fully open-access, and country-specific predictive risk maps only show probabilities of infection prevalence below and above pre-set thresholds where preventive chemotherapy is warranted (e.g. > 50% of school-aged children infected, which demand annual deworming of all school-aged children and adults considered to be at risk) [2]. Starting in late 2006, the European Union (EU)-funded CONTRAST project developed a global database pertaining to NTDs, the GNTD database (http://www.gntd.org) [5]. This open-access database compiled raw survey data from published (i.e. peer-reviewed literature) and unpublished sources (e.g. Ministry of Health reports). It is continuously updated and data can be downloaded as soon as they are entered in the database. In early 2011, the GNTD database consisted of more than 12,000 survey locations for schistosomiasis in Africa [5]. The database has already been utilised for high-spatial resolution schistosomiasis risk mapping and prediction in West Africa [6] and East/southern Africa.

An important drawback of data compilation is the lack of homogeneity and comparability between surveys, such as target population (different age groups), time of survey, diagnostic method employed, among other issues. The GNTD database is populated with schistosomiasis prevalence surveys conducted in schools, as well as in entire communities, involving different, sometimes overlapping age-groups [5]. However, each population sub-group carries a different risk of infection, with school-aged children and adolescence known to carry the highest risk of infection [7,8]. Simple pooling of this type of studies is likely to result in incorrect disease risk estimates.

Schistosomiasis survey data are correlated in space because the disease transmission is driven by environmental factors [9-11]. However, standard statistical modelling approaches assume independence between locations, which could result in inaccurate model estimates [12]. Geostatistical models take into account potential spatial clustering by introducing location-specific random effects and are estimated using Markov chain Monte Carlo (MCMC) simulations [13]. Geostatistical models have been applied on compiled survey data for disease risk prediction, for example in malaria [14-16] and helminth infections, including schistosomiasis [6,17].

Age-heterogeneity of survey data has been addressed in geostatistical modelling by omitting those surveys which consist of particularly heterogeneous age-groups [6,15]. As a result, the number of survey locations included in the analysis is reduced, and hence model accuracy is lowered, especially in regions with sparse data. Gemperli *et al*. [18] used mathematical transmission models to convert age-heterogeneous malaria prevalence data to a common age-independent malaria transmission measure. This approach has been further developed by Gosoniu [19] and Hay *et al*. [16]. To our knowledge, the age-heterogeneity problem has yet to be investigated in schistosomiasis.

In this paper, we developed Bayesian geostatistical models, which take into account age-heterogeneity by incorporating alignment factors to relate schistosomiasis prevalence data from surveys on individuals aged ≤ 20 years with surveys on individuals > 20 years and entire communities. Different models were implemented assuming regional and country-specific alignment factors. The predictive performance of the models was assessed using a suite of model validation approaches. Our analysis is stratified for *Schistosoma haematobium *and *S. mansoni *with a geographical focus on eastern Africa.

## Methods

### Disease data

Prevalence data of *S. haematobium *and *S. mansoni *from 11 countries in eastern Africa were extracted from the GNTD database. We excluded non-direct diagnostic examination techniques, such as immunofluorescence tests, antigen detections or questionnaire data. Hospital-based studies and data on non-representative groups, such as HIV positives, are not part of the GNTD database [5].

The remaining data were split into three groups and stratified for the two *Schistosoma *species according to study type. The three groups correspond to surveys on (i) individuals aged ≤ 20 years, (ii) individuals > 20 years and (iii) entire community surveys. In case a survey contained prevalence data on multiple age groups, we separated the data according to groups (i) and (ii).

Preliminary analyses suggested only weak temporal correlation in the data for either *Schistosoma *species. Hence, spatial models instead of spatio-temporal models were fitted in the subsequent analyses employing the study year only as a covariate. We grouped the study years as follows: surveys conducted (i) before 1980; (ii) between 1980 and 1989; (iii) between 1990 and 1999; and (iv) from 2000 onwards.

### Environmental data

Freely accessible remote sensing data on climatic and other environmental factors were obtained from different sources, as shown in Table 1. Data with temporal variation were obtained from launch until the end of 2009 and summarised as overall averages for the available period. Estimates for day and night temperature were extracted from land surface temperature (LST) data. The normalized difference vegetation index (NDVI) was used as a proxy for vegetation. Land cover categories were restructured into six categories: (i) shrublands and savannah; (ii) forested areas; (iii) grasslands; (iv) croplands; (v) urbanized areas; and (vi) wet areas. Digitized maps of rivers and lakes were combined as a single freshwater map covering the study area. Characteristics on perennial and seasonal water bodies at each survey location were obtained using the spatial join function of ArcMap version 9.2. In addition, the minimum distance between the locations and the closest freshwater source was calculated with the same function.

**Table 1:** Remote sensing data sources^a^

Data type	Source	Date	Temporal resolution	Spatial resolution
LST	MODIS/Terra^1^	2000-2009	8-days	1 km
NDVI	MODIS/Terra^1^	2000-2009	16-days	1 km
Land cover	MODIS/Terra^1^	2001-2004	Yearly	1 km
Rainfall	ADDS^2^	2000-2009	10-days	8 km
Altitude	DEM^3^	-	-	1 km
Water bodies	HealthMapper^4^	-	-	Unknown

^a ^All data accessed on 3 February 2011

^1 ^Moderate Resolution Imaging Spectroradiometer (MODIS) (https://lpdaac.usgs.gov/lpdaac/products/modis_products_table)

^2 ^African Data Dissemination Service (ADDS) (http://earlywarning.usgs.gov/fews/africa/index.php)

^3 ^Digital elevation model (DEM) (http://edc.usgs.gov/#/Find_Data/Products_and_Data_Available/gtopo30/hydro/)

^4 ^HealthMapper database (http://gis.emro.who.int/PublicHealthMappingGIS/HealthMapper.aspx)

All data were used as covariates for modelling. Continuous covariates were categorized based on quartiles in order to account for potential non-linear outcome-predictor relations. Processing and extraction of the climatic and environmental data at the survey locations was performed in ArcMap version 9.2, IDRISI 32 and the Modis Reprojection Tool.

### Geostatistical model formulation and age-alignment

Let *Y_i _*be the number of infected individuals and *N_i _*the number of individuals screened at location *i *(*i *= 1,..., *n*). We assumed that *Y_i _*arises from a Binomial distribution, i.e. *Y_i_*~Bin(*p*_i_,*N*_i_), with probability of infection.*p_i _*We introduced covariates  on the logit scale, such as , where  is the vector of regression coefficients. Unobserved spatial variation can be modelled via additional location-specific random effects, *φ_i_*. We assumed that  arises from a latent stationary Gaussian spatial process,  with correlation matrix *R *modelling geographical dependence between any pairs of locations *i *and *j *via an isotropic exponential correlation function, defined by *R_ij _*= exp(-*ρd_ij_*), where *d_ij _*is the distance between *i *and *j*, ρ a correlation decay parameter and σ^2 ^the spatial variance. A measurement error can also be introduced via location-specific non-spatial random effects, *ε_i_*, such as ε_i_~N(0, *τ*^2^), with non-spatial variance *τ*^2^.

We aligned the risk measured by the different types of studies by incorporating a factor *α_s _*such that *Y_is_*~Bin(*q_i,s,_N_i,s_*), with q*_i,s _*= *α_s_p_i _*and *s *= 1 (surveys with individuals aged ≤ 20 years); *s *= 2 (surveys with individuals aged > 20 years); and *s *= 3 (entire community surveys). School-aged children carry the highest risk of *Schistosoma *infection, and hence many studies focus on this age group. We set α_1 _= 1 in order to use the probability of infection for individuals aged ≤ 20 years as baseline and to align the other groups to this designated baseline.

To complete Bayesian model formulation, we assumed non-informative priors for all parameters. Normal prior distributions with mean 0 and large variance were used for the regression coefficients, . Non-informative Gamma distributions with mean 1 were assumed for the variance parameters, *σ*^2^, *τ*^2 ^and the alignment factors *α*_s_, while a uniform distribution was implemented for the spatial decay parameter *ρ*.

Models were developed in OpenBUGS version 3.0.2 (OpenBUGS Foundation; London, UK) and run with two chains and a burn-in of 5000 iterations. Convergence was assessed by inspection of ergodic averages of selected model parameters and history plots. After convergence, samples of 500 iterations per chain with a thinning of 10 were extracted for each model resulting in a final sample of 1000 estimates per parameter.

### Model types

We implemented four different models, separately for *S. haematobium *and *S. mansoni*. The models varied based on different features. The first feature was the underlying data. Model A only consisted of schistosomiasis prevalence data on individuals aged ≤ 20 years (*s *= 1), while models B-D included data on all three kinds of study types (*s *= 1,2,3). The second feature was the introduction of alignment factors for disease risk modelling. Model C assumed common alignment factors across the entire study region, while model D assumed country-specific alignment factors.

### Model validation

Validation for each model was carried out to identify the model with the highest predictive ability for either *Schistosoma *species and to compare models with and without alignment factors. All models were fitted on a subset of the data (training set) and validated by comparing the posterior median of the predicted risk  with the observed risk *P_j _*for the remaining set of the data (test set, *j *= 1,...,*m, m *<*n*). The test set consisted of 20% of the locations from the dataset on individuals aged ≤ 20 years and was congruent over all models.

Comparisons of predicted *vs*. observed risk were based on three different validation approaches. Mean absolute errors (MAE) calculate the absolute difference between observed and predicted schistosomiasis risk by . An alternative way to quantify divergences in the predictions to the observed data is the χ^2 ^measure, defined as . The best predicting model based on these two methods is the model with smallest MAE and χ^2 ^estimates and therefore with predictions closest to the observed values.

The proportion of the test data being correctly predicted within the *q*-th Bayesian credible interval (BCI_q_) of the posterior predictive distribution is calculated by , with *q *= 50%, 70%, 90% and 95%. For this approach, the best performing model contains most test locations within BCIs of smallest width.

## Results

### Schistosomiasis prevalence data

**Figure 1:**
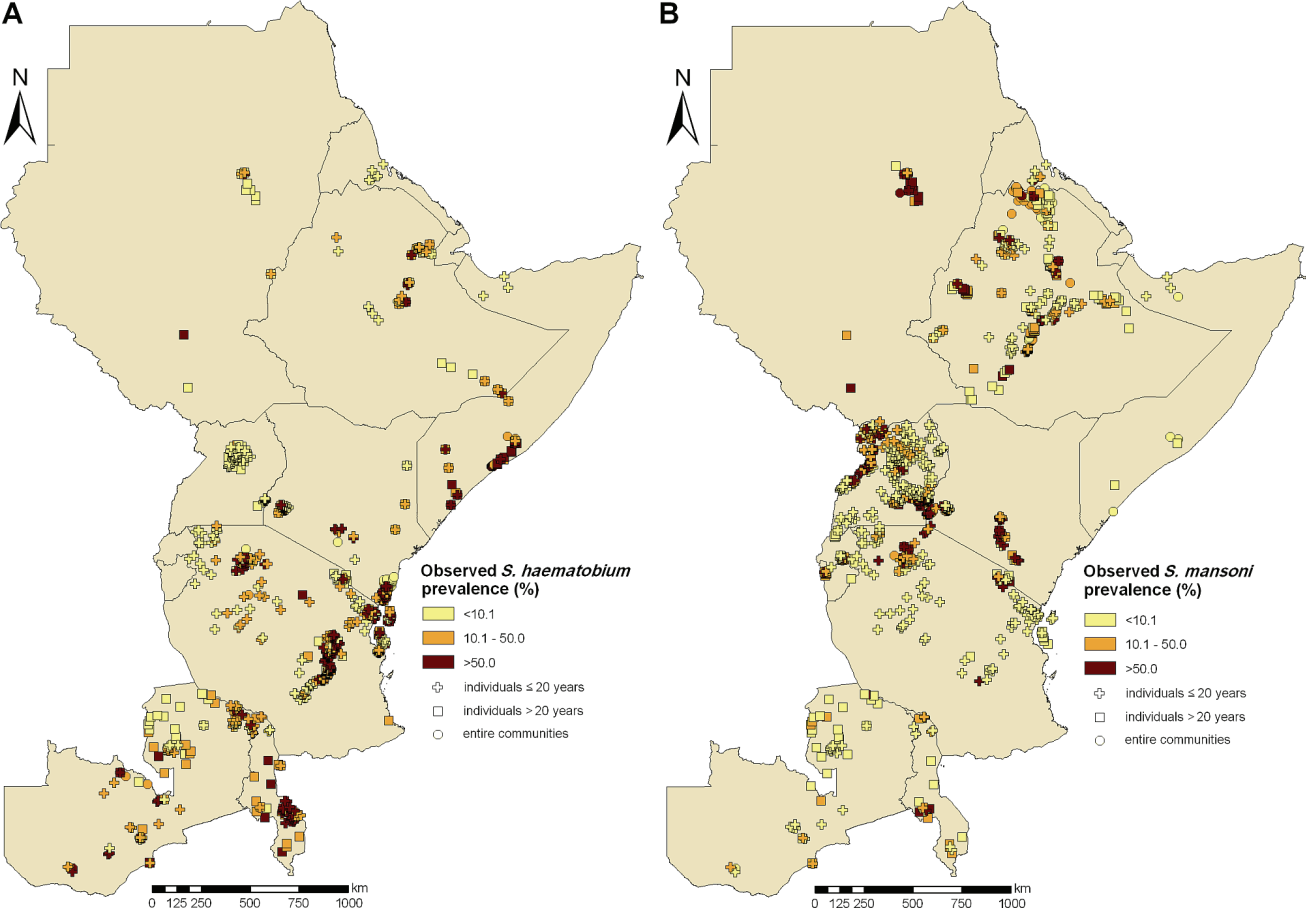
**Compiled prevalence data of *Schistosoma haematobium *(A) and *S. mansoni *(B) across eastern Africa**. Prevalence data are stratified by three different age groups.

Figure 1 shows the distribution of the observed schistosomiasis prevalence data over the study region, stratified by study type. An overview of the amount of observed data and mean prevalence levels per country for either *Schistosoma *species, stratified by survey period and diagnostic methods, is given in Table 2. Some countries (e.g. Kenya and Tanzania), contain large numbers of survey locations, while other countries, such as Burundi, Eritrea, Rwanda, Somalia and Sudan, are not well covered. Burundi and Rwanda do not include any locations for *S. haematobium*, and Rwanda contains only four surveys on individuals aged > 20 years for *S. mansoni*. As expected, there were more surveys carried out with individuals aged ≤ 20 years than surveys focussing on adult populations or entire communities.

**Table 2:** Overview of schistosomiasis surveys, stratified by survey year, diagnostic method, country and age group

	Age*	Survey year				Diagnostic^†^		TOTAL
		< 1980	1980- 1989	1990- 1999	2000- 2009			
***S. haema-tobium***						**UC**	**RS**	
Burundi	1	-	-	-	-	-	-	-
	2	-	-	-	-	-	-	-
	3	-	-	-	-	-	-	-
Eritrea	1	0.0 (4)	-	-	-	0.0 (4)	-	0.0 (4)
	2	0.0 (1)	-	-	-	0.0 (1)	-	0.0 (1)
	3	0.0 (2)	-	-	-	0.0 (2)	-	0.0 (2)
Ethiopia	1	17.0 (7)	30.5 (6)	25.2 (18)	-	22.7 (10)	22.2 (17)	24.4 (31)
	2	15.0 (6)	27.6 (5)	16.4 (12)	-	20.7 (11)	17.9 (6)	18.5 (23)
	3	35.0 (3)	29.5 (4)	74.1 (1)	-	38.4 (5)	-	37.1 (8)
Kenya	1	21.0 (65)	52.8 (15)	54.0 (30)	40.6 (2)	34.4 (109)	37.5 (3)	34.5 (112)
	2	22.7 (6)	49.6 (7)	30.3 (3)	-	34.9 (15)	50.7 (1)	35.9 (16)
	3	24.8 (7)	14.0 (25)	64.9 (2)	45.8 (6)	23.2 (40)	-	23.3 (40)
Malawi	1	22.6 (2)	-	55.5 (40)	-	21.5 (6)	59.3 (36)	53.9 (42)
	2	48.2 (7)	34.3 (8)	75.0 (1)	31.6 (5)	36.4 (17)	31.5 (1)	40.2 (21)
	3	31.4 (1)	-	-	-	31.4 (1)	-	31.4 (1)
Rwanda	1	-	-	-	-	-	-	-
	2	-	-	-	-	-	-	-
	3	-	-	-	-	-	-	-
Somalia	1	39.4 (11)	-	-	-	39.4 (11)	-	39.4 (11)
	2	55.5 (22)	87.1 (1)	-	-	56.9 (23)	-	56.9 (23)
	3	53.1 (21)	-	-	-	53.1 (21)	-	53.1 (21)
Sudan	1	5.2 (1)	45.3 (3)	-	-	31.2 (2)	-	35.2 (4)
	2	-	1.8 (7)	-	53.0 (3)	10.7 (8)	-	17.2 (10)
	3	1.8 (2)	20.6 (2)	-	-	20.6 (2)	-	11.2 (4)
Tanzania	1	41.5 (48)	39.9 (173)	30.2 (12)	20.0 (84)	30.6 (173)	59.8 (73)	34.5 (317)
	2	45.5 (41)	29.2 (9)	20.0 (8)	10.0 (2)	39.1 (47)	-	38.5 (60)
	3	47.1 (16)	30.3 (3)	-	28.4 (3)	42.2 (20)	-	42.3 (22)
Uganda	1	42.5 (2)	-	-	0.6 (36)	2.8 (38)	-	2.8 (38)
	2	9.8 (14)	-	-	0.8 (3)	8.7 (16)	-	8.2 (17)
	3	33.3 (2)	-	-	3.0 (2)	18.1 (4)	-	18.1 (4)
Zambia	1	28.2 (22)	30.5 (11)	29.6 (32)	49.2 (4)	31.0 (38)	29.8 (31)	30.5 (69)
	2	13.7 (30)	35.1 (6)	22.9 (5)	13.3 (1)	17.9 (42)	-	17.9 (42)
	3	17.0 (3)	62.4 (3)	35.5 (1)	32.5 (3)	37.1 (10)	-	37.1 (10)

TOTAL	1	28.8 (162)	40.1 (208)	42.4 (132)	15.7 (126)	28.6 (391)	49.4 (160)	32.8 (628)
	2	33.1 (127)	31.0 (43)	22.0 (29)	25.2 (14)	30.5 (180)	26.7 (8)	30.6 (213)
	3	40.3 (57)	21.3 (37)	59.8 (4)	33.1 (14)	34.2 (105)	-	33.8 (112)

***S. mansoni***						**KK**	**SC**	
Burundi	1	-	16.4 (12)	38.3 (3)	-	20.8 (15)	-	20.8 (15)
	2	-	20.8 (19)	44.1 (2)	-	23.0 (21)	-	23.0 (21)
	3	-	19.8 (8)	50.5 (2)	-	25.9 (10)	-	25.9 (10)
Eritrea	1	12.5 (4)	-	-	-	-	12.5 (4)	12.5 (4)
	2	10.0 (1)	-	-	-	-	10.0 (1)	10.0 (1)
	3	41.7 (2)	-	-	-	-	41.7 (2)	41.7 (2)
Ethiopia	1	2.7 (27)	25.0 (23)	28.0 (51)	33.0 (4)	30.1 (69)	6.9 (36)	22.2 (105)
	2	25.7 (15)	18.6 (93)	18.4 (36)	41.8 (7)	18.8 (100)	23.2 (50)	20.3 (151)
	3	4.6 (9)	16.0 (8)	21.1 (62)	36.3 (4)	26.4 (28)	16.0 (55)	19.5 (83)
Kenya	1	18.6 (48)	72.8 (9)	74.3 (18)	53.8 (15)	68.4 (43)	8.7 (39)	41.0 (90)
	2	49.2 (7)	75.2 (7)	36.0 (5)	33.3 (1)	65.4 (14)	28.1 (6)	54.2 (20)
	3	30.7 (15)	71.7 (9)	23.6 (2)	-	69.8 (12)	22.5 (14)	44.3 (26)
Malawi	1	19.4 (1)	37.5 (2)	20.3 (6)	-	30.8 (7)	0.4 (2)	24.0 (9)
	2	17.9 (6)	36.8 (6)	-	-	35.3 (8)	-	27.3 (12)
	3	-	-	-	-	-	-	25.9 (21)
Rwanda	1	-	-	-	-	-	-	-
	2	-	4.6 (4)	-	-	-	-	4.6 (4)
	3	-	-	-	-	-	-	-
Somalia	1	0.0 (3)	-	-	-	-	0.0 (3)	0.0 (3)
	2	0.0 (2)	-	-	-	-	0.0 (2)	0.0 (2)
	3	0.0 (3)	0.0 (2)	-	-	-	0.0 (5)	0.0 (5)
Sudan	1	61.7 (4)	61.5 (4)	-	-	61.9 (6)	-	61.6 (8)
	2	62.3 (4)	64.9 (8)	47.0 (1)	56.3 (3)	65.8 (15)	0.3 (1)	61.7 (16)
	3	41.0 (2)	52.3 (4)	-	-	50.5 (5)	-	48.5 (6)
Tanzania	1	20.3 (27)	25.3 (4)	30.8 (7)	8.9 (77)	39.6 (17)	21.7 (21)	13.5 (115)
	2	22.2 (26)	12.0 (1)	25.6 (3)	38.6 (5)	46.4 (6)	26.3 (18)	24.8 (35)
	3	27.3 (14)	11.6 (1)	44.1 (3)	44.4 (3)	49.8 (5)	27.3 (14)	31.4 (21)
Uganda	1	48.0 (5)	48.7 (3)	14.5 (6)	22.1 (263)	22.2 (272)	48.0 (5)	22.7 (277)
	2	24.2 (17)	56.3 (3)	66.7 (5)	40.8 (12)	49.6 (20)	20.9 (12)	37.9 (37)
	3	41.7 (7)	47.8 (4)	45.0 (5)	55.8 (12)	50.5 (21)	47.0 (6)	48.3 (28)
Zambia	1	2.9 (16)	5.7 (7)	71.0 (1)	33.2 (1)	36.1 (4)	4.3 (10)	7.7 (25)
	2	8.4 (30)	0.0 (1)	-	41.7 (1)	41.7 (1)	8.1 (31)	9.2 (32)
	3	5.0 (2)	9.5 (1)	60.1 (1)	33.5 (1)	34.4 (3)	5.0 (2)	22.6 (5)

TOTAL	1	16.6 (135)	31.8 (64)	36.7 (92)	20.7 (360)	29.5 (433)	11.5 (120)	23.2 (651)
	2	21.7 (108)	25.3 (142)	26.8 (52)	42.0 (29)	31.7 (185)	19.1 (121)	25.8 (331)
	3	25.0 (54)	28.6 (243)	24.9 (75)	49.1 (20)	41.7 (84)	19.9 (98)	29.7 (186)

Overall and country-specific mean observed prevalence data (expressed in %) are given, along with number of survey locations (in brackets) for both *S. haematobium *and *S. mansoni*.

* 1, individuals aged ≤ 20 years; 2, individuals aged > 20 years; 3, entire communities

^† ^UC, urine concentration by sedimentation, filtration or centrifugation; RS, reagent strips; KK, Kato-Katz thick smear method; SC, stool concentration methods. Results for surveys with missing diagnostic methods were omitted.

The mean prevalence per country for surveys on individuals aged ≤ 20 years varies between 0% (Eritrea) and 53.9% (Malawi) for *S. haematobium *and between 0% (Somalia) and 61.6% (Sudan) for *S. mansoni*. We found an overall mean prevalence of *S. haematobium *and *S. mansoni *of 32.8% and 23.2%, respectively. Community surveys usually showed higher mean prevalence levels. However, the survey locations might not be the same among the different types of studies and therefore the observed prevalence levels are not directly comparable.

Two-third of the *S. haematobium *survey data were obtained before the 1990s (66.5%), while few surveys were compiled from 2000 onwards (16.2%). On the other hand, *S. mansoni *surveys were mainly conducted in the 1980s (32.7%) and from 2000 onwards (29.8%), whereas only 15.9% of the surveys were carried out in the 1990s. The distribution of surveys within the different time periods varies from country to country and between the two *Schistosoma *species. While some countries (e.g. Eritrea and Somalia) only have surveys for one or two periods, other countries (e.g. Kenya, Tanzania and Zambia) are well covered over time. The data also vary in the diagnostic methods. For example, even though 67.4% of the *S. mansoni *surveys with known diagnostic methods employed the Kato-Katz thick smear method, in Somalia and Eritrea only stool concentration methods (e.g. Ritchie technique or ether-concentration technique) were used.

### Model validation

For *S. haematobium*, model validation based on the MAE measure (Table 3) showed no difference between disease risk modelling on individuals aged ≤ 20 years (model A) and unaligned modelling of all three survey types (model B), while the χ^2 ^measure led to improved predictions. The introduction of regional alignment factors in spatial modelling based on all survey types (model C) further enhanced model predictive ability based on the MAE and χ^2 ^measures. Model D, including country-specific alignment factors, showed similar predictive performance as model B. Validation based on different BCIs demonstrated that the proportion of correctly predicted test locations was similar among all models. Model A predicted most test locations correctly within the 95% BCI, while model C was superior for 50% BCIs and model D for 70% BCIs. Regardless of the model used, average BCI widths were comparable.

**Table 3:** Model validation results based on MAE, χ^2 ^measure and BCIs

	Model A	Model B	Model C	Model D
Age groups	≤ 20 years	All	All	All
Alignment	-	-	Regional	Country
***S. haematobium***				
MAE	16.4	16.7	15.5	16.5
χ^2^	126.4	95.7	72.6	96.6
50% BCI (width of BCI)	39.7 (24.8)	41.1 (24.3)	42.1 (25.2)	38.1 (24.5)
70% BCI (width of BCI)	57.1 (36.9)	57.1 (36.3)	61.1 (37.7)	61.9 (36.5)
90% BCI (width of BCI)	75.4 (54.1)	75.4 (53.5)	75.4 (54.7)	75.4 (53.9)
95% BCI (width of BCI)	84.9 (61.0)	81.0 (60.4)	79.4 (61.2)	80.2 (60.6)

***S. mansoni***				
MAE	11.5	11.3	11.0	11.5
χ^2^	48.3	46.8	39.7	43.1
50% BCI (width of BCI)	41.5 (18.5)	34.6 (16.1)	36.9 (15.2)	40.0 (16.4)
70% BCI (width of BCI)	57.7 (29.0)	60.8 (25.4)	60.8 (24.7)	60.8 (26.0)
90% BCI (width of BCI)	80.0 (47.6)	79.2 (41.3)	80.0 (41.6)	81.5 (44.0)
95% BCI (width of BCI)	88.5 (56.5)	84.6 (49.6)	83.8 (50.2)	83.8 (52.7)

BCI, Bayesian credible interval; MAE, mean absolute error.

For *S. mansoni*, model predictive performance in terms of MAE and χ^2 ^measures was best for model C, followed by models B and D. The differences among the models for the BCI method were small and not consistent between the examined BCIs. For example, at 70% BCI, model A included least of the test locations, while at 95% BCI, this model correctly predicted most of the test locations but the averaged width of the BCI was widest.

### Alignment factors

Regional and country-specific schistosomiasis risk alignment factors for *S. haematobium *and *S. mansoni *are presented in Table 4. Some countries had insufficient data, and hence country-wide alignment factors could not be estimated. A mean regional alignment factor of 0.83 (95% BCI: 0.81-0.85) confirmed that the risk of *S. haematobium *in individuals aged ≤ 20 years is greater than in individuals > 20 years. *S. haematobium *risk estimation from entire community survey was related to the risk of individuals aged ≤ 20 years with 0.91 (95% BCI: 0.90-0.93). Mean country-specific alignment factors varied from 0.62 (Ethiopia) to 1.26 (Zambia) among individuals > 20 years and from 0.79 (Ethiopia) to 1.06 (Zambia) in entire communities. In Ethiopia and Sudan, the country-specific alignment factors were significantly smaller than the overall alignment factor, whereas in Somalia and Zambia, country-specific factors were significantly larger.

**Table 4:** Overview of observed data and alignment factor results, stratified by country, *Schistosoma *species and age group

		*S. haematobium*			*S. mansoni*		
	Age*	N	p	Alignment factor	N	p	Alignment factor
Burundi	1	0	-	1.00	15	20.8	1.00
	2	0	-	-	21	23.0	0.78 (0.71, 0.87)
	3	0	-	-	10	25.9	0.84 (0.76, 0.93)
Eritrea	1	4	0.0	1.00	4	12.5	1.00
	2	1	0.0	-	1	10.0	-
	3	2	0.0	-	2	41.7	-
Ethiopia	1	31	24.4	1.00	105	22.2	1.00
	2	23	18.5	0.62 (0.55, 0.68)	151	20.3	0.71 (0.70, 0.73)
	3	8	37.1	0.79 (0.72, 0.87)	83	19.5	0.85 (0.83, 0.88)
Kenya	1	112	34.5	1.00	90	41.0	1.00
	2	16	35.9	0.84 (0.79, 0.89)	20	54.2	1.09 (1.05, 1.13)
	3	40	23.2	0.86 (0.83, 0.89)	26	44.3	1.02 (0.99, 1.05)
Malawi	1	42	53.9	1.00	9	24.0	1.00
	2	21	40.2	0.86 (0.82, 0.92)	12	27.3	-
	3	1	31.4	-	0	-	-
Rwanda	1	0	-	1.00	0	-	1.00
	2	0	-	-	4	4.6	-
	3	0	-	-	0	-	-
Somalia	1	11	39.4	1.00	3	0.0	1.00
	2	23	56.9	1.05 (0.95, 1.18)	2	0.0	-
	3	21	53.1	1.02 (0.94, 1.12)	5	0.0	-
Sudan	1	4	35.2	1.00	8	61.6	1.00
	2	10	17.2	0.69 (0.64, 0.74)	16	61.7	1.02 (0.94, 1.10)
	3	4	11.2	-	6	48.5	1.00 (0.95, 1.06)
Tanzania	1	317	34.5	1.00	115	13.5	1.00
	2	60	38.5	0.84 (0.82, 0.86)	35	24.8	1.18 (1.12, 1.24)
	3	22	42.3	0.94 (0.91, 0.96)	21	31.4	1.13 (1.08, 1.17)
Uganda	1	38	2.8	1.00	277	22.7	1.00
	2	17	8.2	0.89 (0.77, 1.03)	37	37.9	1.06 (1.01, 1.11)
	3	4	18.1	-	28	48.3	1.01 (0.96, 1.04)
Zambia	1	69	30.5	1.00	25	7.7	1.00
	2	42	17.9	1.26 (1.08, 1.43)	32	9.2	0.64 (0.49, 0.89)
	3	10	37.1	1.06 (0.99, 1.13)	5	22.6	-
TOTAL	1	628	32.8	1.00	651	23.2	1.00
	2	213	30.6	0.83 (0.81, 0.85)	331	25.8	0.94 (0.92, 0.96)
	3	112	33.8	0.91 (0.90, 0.93)	186	29.7	0.96 (0.95, 0.98)

Overall and country-specific number of survey locations (N), mean observed prevalence (p) and alignment factor results (with 95% BCI given in brackets) per age group and *Schistosoma *species.

* 1, individuals aged ≤ 20 years; 2, individuals aged > 20 years; 3, entire communities.

For *S. mansoni*, the mean regional alignment factor among individuals aged > 20 years was 0.94 (95% BCI: 0.92-0.96), while country-specific estimates varied from 0.64 (Zambia) to 1.18 (Tanzania). In community surveys, the regional alignment factor was 0.96 (95% BCI: 0.95-0.98) with country-specific alignment factors between 0.84 (Burundi) and 1.13 (Uganda). Significantly smaller country-specific alignment factors compared to the overall alignment factor were found in Burundi, Ethiopia and Zambia, while significantly larger factors were obtained for Kenya, Tanzania and Uganda.

The regional alignment factor estimates for *S. haematobium *compared to *S. mansoni *are much lower, e.g. 17% risk reduction for individuals aged > 20 years *vs*. 6% risk reduction. This relation is also found in country-specific estimates, except for Zambia.

## Discussion

In this study, we derived factors to align schistosomiasis prevalence estimates from age-heterogeneous surveys across an ensemble of 11 countries in eastern Africa. We found correction factors that are significantly different from 1. As a result, geostatistical model-based predictions from school-based and community-based surveys are further enhanced. The estimates of the regional alignment factors confirm that individuals aged ≤ 20 years are at a higher risk of a *Schistosoma *infection than adults [7,8,20]. Interestingly, the alignment factor estimates for *S. haematobium *were slightly lower than those for *S. mansoni*. This finding might be explained by differences in the age-prevalence curves between the two species. *S. haematobium *prevalence usually peaks in the age group 10-15 years [21], while the peak of *S. mansoni *prevalence occurs somewhat later, up to the age of 20 years [22]. Consequently, there is a larger difference in infection risk between children and adults for *S. haematobium *compared to *S. mansoni*. Additionally, the peak of *S. mansoni *prevalence might be further shifted towards older age groups due to the so-called peak shift. Indeed, it has been shown that the peak of infection prevalence is more flat and reaches its maximum in older age groups if transmission is low-to-moderate, while prevalence peaks are higher and they are observed at a younger mean age if transmission is high [7]. Several African countries have implemented large-scale preventive chemotherapy programmes against schistosomiasis [3,23]. These programmes reduced schistosomiasis-related morbidity [24] and might have had some impact on transmission [25,26]. It is therefore conceivable that the peak of *Schistosoma *infection might slightly shift to older age groups. It should also be noted that, disparities in the spatial risk distribution of the two *Schistosoma *species and in the implementation of control strategies in these areas could have led to differences in the alignment factors.

Considerable differences between country-specific alignment factors and prevalence ratios based on the raw data were found for Ethiopia, Tanzania, Uganda and Zambia in *S. haematobium*, and for Burundi and Zambia in *S. mansoni*. These differences are mainly due to the spatial distribution of the survey locations, which vary between age groups. For example, surveys focussing on individuals aged ≤ 20 years are located in central and eastern Zambia, while surveys on individuals > 20 years in Zambia are mainly located in the north of the country. The north is characterised by lower schistosomiasis transmission risk. Therefore, the crude prevalence ratio between the two groups is artificially small, while the alignment factor, which is based on the predicted prevalence risk in this area, is much higher.

Model validation showed that regional alignment factors improved predictive performance of the models for both *Schistosoma *species, however, country-specific alignment factors did not further improve the models. The predictive performance of the model with regional factors was good, as 79.4% and 83.8% of the test locations were correctly predicted within 95% BCIs for *S. haematobium *and *S. mansoni*, respectively. All models estimated relatively wide BCIs, indicating large variation in the data that could not be explained by the model covariates. Socioeconomic and health system factors might play a role in the spatial distribution of schistosomiasis, however these data do not exist at high spatial distribution for the entire study area, and hence could not be used for model fit and prediction. Part of the variation might have arisen by the model assumptions of stationarity and isotropy and the heterogeneity in the diagnostic methods.

The proposed alignment factor approach is scaling the predicted prevalence of schistosomiasis and leads to an easy interpretation of the parameters. In addition, it allows defining meaningful prior distributions, and hence resulting in better model convergence. An alternative way to include age in the models is to introduce age as a covariate. This approach is scaling the odds instead of the prevalence. Preliminary analyses preformed by the authors, on the same data using age as covariate, resulted in serious model convergence problems, leading to the implementation of age alignment factors as proposed in this manuscript.

A limitation of our work is the assumption of constant disease risk within each age group. This is not true especially for school-aged children for whom the schistosomiasis risk reaches a maximum at around 11-14 years. A more rigorous model formulation should take into account the age-prevalence curve and standardise the surveys using a mathematical description of this curve. Raso *et al*. [27] derived a Bayesian formulation of the immigration-death model to obtain age-specific prevalence of *S. mansoni *from age-prevalence curves. We are currently exploring geostatistical models, coupled with mathematical immigration-death models, to fully consider the age-dependence of the schistosomiasis risk.

## Conclusions

We have shown that age-alignment factors should be included to improve prevalence estimates of population-based risk of schistosomiasis, especially for large-scale modelling and prediction efforts. Indeed, large-scale modelling cannot be achieved without compilation of primarily historical survey data assembled over large study areas using different study designs and age groups. The proposed alignment factor approach can be used to relate the most frequent survey types, i.e. studies focussing on individuals aged ≤ 20 years (mainly school surveys) with studies on individuals aged > 20 years and entire communities. Un-aligned survey compilation leads to imprecise disease risk estimates and potentially wrong recommendations to decision makers for the implementation of control activities and subsequent monitoring and evaluation.

## List of abbreviations

ADDS: African data dissemination service; BCI: Bayesian credible interval; DEM: digital elevation model; EU: European Union; GAHI: Global atlas of helminth infections; GNTD database: Global neglected tropical disease database; LST: Land surface temperature; MAE: Mean absolute error; MCMC: Markov chain Monte Carlo; NDVI: Normalized difference vegetation index; NTD: Neglected tropical disease.

## Competing interests

The authors declare that they have no competing interests.

## Authors' contributions

NS participated in data acquisition and design of the study, was responsible for data analysis and drafted the manuscript. JU participated in the design of the study, helped interpreting the results and revised the manuscript. PV designed the study, contributed to data analysis and was involved in drafting the manuscript. All authors read and approved the manuscript prior to submission.
